# *EGFR* and *KRAS* mutations in lung carcinomas in the Dutch population: increased *EGFR* mutation frequency in malignant pleural effusion of lung adenocarcinoma

**DOI:** 10.1007/s13402-012-0078-4

**Published:** 2012-04-12

**Authors:** Alexander J. J. Smits, J. Alain Kummer, John W. J. Hinrichs, Gerarda J. M. Herder, Karen C. Scheidel-Jacobse, N. Mehdi Jiwa, T. Emiel G. Ruijter, Peet T. G. A. Nooijen, Monica G. Looijen-Salamon, Marjolijn J. L. Ligtenberg, Frederik B. Thunnissen, Daniëlle A. M. Heideman, Roel A. de Weger, Aryan Vink

**Affiliations:** 1 Department of Pathology, St. Antonius Hospital, Nieuwegein, The Netherlands; 2 Department of Pathology, University Medical Center Utrecht, PO Box 85500, 3508 GA Utrecht, The Netherlands; 3 Department of Pulmonary Diseases, St. Antonius Hospital, Nieuwegein, The Netherlands; 4 Department of Pathology, Medical Center Alkmaar, Alkmaar, The Netherlands; 5 Department of Pathology, Rijnstate Hospital, Arnhem, The Netherlands; 6 Department of Pathology, Jeroen Bosch Hospital, ’s Hertogenbosch, The Netherlands; 7 Department of Pathology, Radboud University Nijmegen Medical Centre, Nijmegen, The Netherlands; 8 Department of Pathology, VU University Medical Center, Amsterdam, The Netherlands

**Keywords:** Non-small cell lung cancer, Adenocarcinoma, *EGFR*, *KRAS*, Metastasis, Pleural effusion

## Abstract

**Background:**

Frequencies of *EGFR* and *KRAS* mutations in non-small cell lung cancer (NSCLC) have predominantly been determined in East Asian and North American populations, showing large differences between these populations. The aim of the present study was to determine the frequency of *EGFR* and *KRAS* mutations in NSCLC in the West European Dutch population in primary carcinomas and different metastatic locations.

**Methods:**

*EGFR* (exons 19, 20 and 21) and *KRAS* (exons 2 and 3) mutation test results of NSCLC samples of patients in 13 hospitals were collected. The tests were performed on paraffin-embedded tissue or cytological material of primary and metastatic lung carcinomas.

**Results:**

*EGFR* mutations were detected in 71/778 (9.1 %) tested patients; in 66/620 (10.6 %) adenocarcinomas. *EGFR* mutations were significantly more often detected in female than in male patients (13.4 % vs. 5.5 %, *p* < 0.001). *KRAS* mutations were found in 277 out of 832 (33.3 %) tested patients; in 244/662 (36.9 %) adenocarcinomas. A significantly increased frequency of *EGFR* mutations was observed in patients with malignant pleural/pericardial effusions (26.5 %; odds ratio (OR) 2.80, 95 % confidence interval (CI) 1.22–6.41), whereas the frequency of *KRAS* mutations was significantly decreased (18.8 %; OR 0.35, 95 % CI 0.14–0.86).

**Conclusions:**

In the investigated Dutch cohort, patients with malignant pleural/pericardial effusion of lung adenocarcinoma have an increased frequency of EGFR mutations. The overall frequency of *EGFR* mutations in lung adenocarcinomas in this West European population is within the frequency range of North American and South European populations, whereas *KRAS* mutation frequency is higher than in any population described to date.

## Introduction

Non-small-cell lung cancer (NSCLC) often presents at an advanced stage with no options for curative treatment. Response to chemotherapy is rather poor, resulting in a median overall survival of approximately 1 year. New therapies, targeting specific signaling pathways, have been developed over the last years. These include the small-molecule tyrosine kinase inhibitors (TKIs) erlotinib and gefitinib, blocking signaling through the epidermal growth factor receptor (EGFR). Treatment with these TKIs seems to be especially effective in tumors with activating mutations in the *EGFR* gene, whereas tumors harboring activating mutations in the *KRAS* gene do not respond to this treatment [1].

The frequency of *EGFR* mutations has been studied most extensively in East Asian populations, where it varies from 36.4 to 66.3 % in adenocarcinoma (ADC) [2, 3]. Studies in North American and South European populations show considerably lower numbers ranging from 6.0 to 14.0 % [4, 5]. For *KRAS* the situation is reversed, with a low mutation frequency of 2.3 to 9.4 % in East Asian [3, 6] and a higher frequency of 11.7 to 31.0 % in North American and South European populations [7, 8]. However, for the West European population the frequency of *EGFR* and *KRAS* mutations has not been studied in large numbers of patients.

In East Asian and South European populations it has been demonstrated that certain patient characteristics are associated with an increased *EGFR* mutation rate. *EGFR* mutations in these populations are associated with female patients, patients who have never smoked and patients with an adenocarcinoma [3, 9, 10]. In these studies *EGFR* mutations are usually studied in tissue from the primary lung tumor. Although the association between patient characteristics and mutation status has been investigated extensively, the association between different metastatic locations and *EGFR* status has remained relatively underexplored.

The aim of the present study was to determine the frequency of *EGFR* and *KRAS* mutations in the Dutch population in both primary NSCLCs and different metastatic locations.

## Methods

### Patients and tumor samples

We collected data of all NSCLC samples used for routine diagnostic *EGFR* and *KRAS* mutation analysis from 13 hospitals in the Netherlands from January 2008 to April 2011. The participating hospitals were St. Antonius Hospital, Nieuwegein; University Medical Center Utrecht; Rijnstate Hospital, Arnhem; Medical Center Alkmaar; Jeroen Bosch Hospital, ‘s Hertogenbosch; Radboud University Nijmegen Medical Center; Zuwe Hofpoort Hospital, Woerden; Slingeland Hospital, Doetinchem; Gemini Hospital, Den Helder; Hospital Gelderse Vallei, Ede; Hospital Bernhoven, Oss and Veghel; Maas Hospital Pantein, Boxmeer; and Elkerliek Hospital, Helmond. Mutation analysis was centrally performed in four Pathology Departments (St. Antonius Hospital, Nieuwegein; University Medical Center Utrecht; VU University Medical Center, Amsterdam; and Radboud University Nijmegen Medical Center).

The results were obtained using formalin-fixed paraffin-embedded tissue or cytological slides of primary lung tumors and metastases. The patients’ smoking status was included for a subgroup of patients whose samples were analyzed at St. Antonius Hospital, Nieuwegein and University Medical Center, Utrecht. Patients were categorized as never smokers (<100 lifetime cigarettes), former smokers (≥1 year since cessation), or current smokers (still smoking or <1 year since cessation). When available, the number of packyears was included in the smoking history.

### Ethics statement

Specific approval of the ethics committee was not necessary for this study, since all mutation analyses were part of the routine diagnostic procedure and all patient and tumor characteristics were collected anonymously.

### *EGFR* and *KRAS* mutation analysis

Two methods were used for *EGFR* and *KRAS* mutation analysis. In most cases mutation analysis was performed by polymerase chain reaction followed by sequencing of the *EGFR* (exons 19, 20, and 21) and *KRAS* (exons 2 and 3) genes, using GenBank Accession Numbers NM_005228.3 and NM_004985.3 as a reference. The samples of the patients from the hospitals in Utrecht, Alkmaar, and ‘s Hertogenbosch were tested using high resolution melting analysis, followed by sequencing only if the obtained melting curve was abnormal, as described previously [11].

### Statistical analysis

Statistical calculations were performed using SPSS software (version 15.0). Associations between *EGFR* and *KRAS* status and patient and tumor characteristics were analyzed using the Fisher’s exact test. Normal distribution of age and number of packyears was tested using the Kolmogorov-Smirnov test and means were compared using the Mann–Whitney test or the *T* test when appropriate. Binary logistic regression analysis was used to compare the mutation frequency of different metastatic sites to the group of primary tumors. Odds ratio (OR) and 95 % confidence interval (CI) were calculated. In all tests, two-sided p-values of less than 0.05 were considered statistically significant.

## Results

### *EGFR* mutations

*EGFR* mutation status was determined in 816 samples, 791 of which had an interpretable result. The samples consisted of 655 cases of ADC, 42 cases of squamous cell carcinoma (SCC), and 119 others (102 large cell carcinomas, 8 sarcomatoid carcinomas, 5 adenosquamous carcinomas, 4 unspecified). Of these samples, 462 were derived from primary tumors and 342 from different metastatic sites. For 12 pleural biopsies/excisions, distinction between a primary tumor and a metastasis was not possible. The samples were derived from 803 individual patients, of whom there was an interpretable result in 778 patients. For 13 patients the mutation status of both their primary tumors and a metastasis was determined. These patients were considered *EGFR* mutation positive if either the primary tumor or the metastasis was tested positive. *EGFR* mutations were detected in 71 (9.1 %) individual patients, 66 of whom had a diagnosis of ADC (frequency in ADC 10.6 %). The other 5 mutations were found in 3 large cell carcinomas, one adenosquamous carcinoma, and one sarcomatoid carcinoma (subtype spindle cell carcinoma). *EGFR* mutations were significantly more often observed in ADC than in SCC or other tumor types (10.6 %, 0 %, and 4.3 % respectively, *p* = 0.006) and more frequently in female than in male patients (13.4 % vs. 5.5 %, *p* < 0.001) (Table 1). The smoking history could be obtained for 288 patients with a known *EGFR* mutation status. The *EGFR* mutation frequency was significantly higher in never smokers compared to former and current smokers (48.3 % vs. 8.5 % vs. 4.9 % respectively, *p* < 0.001). Within the group of former and current smokers, the average number of packyears smoking history was significantly lower in *EGFR* mutation positive compared to *EGFR* mutation negative patients (23.6 vs. 35.6, *P* = 0.031).

**Table 1:** Frequency of *EGFR* and *KRAS* mutations

	*EGFR* mutated N (%)	*EGFR* wild type N (%)	p-value	*KRAS* mutated N (%)	*KRAS* wild type N (%)	p-value
Total	71 (9.1)	707 (90.9)		277 (33.3)	555 (66.7)	
Mean age years ± sem	61.6 ± 1.28	62.7 ± 0.39	0.347	61.1 ± 0.63	62.8 ± 0.45	0.014
Sex						
Male	23 (5.5)	398 (94.5)	<0.001	137 (30.6)	310 (69.4)	0.090
Female	48 (13.4)	309 (86.6)		140 (36.4)	245 (63.6)	
Smoking history						
Never	14 (48.3)	15 (51.7)	<0.001	3 (12.0)	22 (88.0)	0.044
Former	10 (8.5)	107 (91.5)		37 (33.9)	72 (66.1)	
Current	7 (4.9)	135 (95.1)		56 (36.6)	97 (63.4)	
Mean no. of packyears^a^	23.6	35.6	0.031	30.7	29.9	0.808
Histology						
ADC	66 (10.6)	554 (89.4)	0.006	244 (36.9)	418 (63.1)	<0.001
SCC	0 (0)	41 (100)		1 (2.5)	39 (97.5)	
Other	5 (4.3)	112 (95.7)		32 (24.6)	98 (75.4)	

*sem* standard error of the mean; *ADC* adenocarcinoma; *SCC* squamous cell carcinoma

^a^For former and current smokers only

**Fig. 1:**
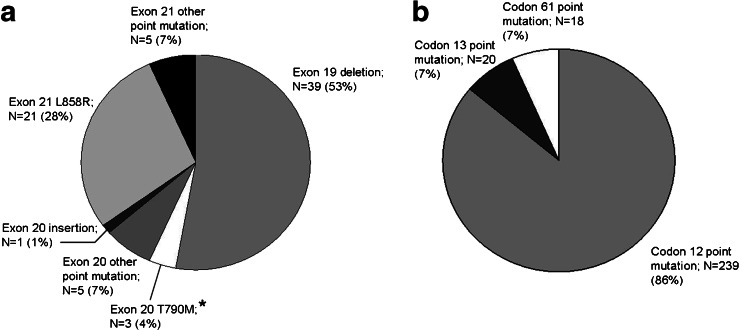
Distribution of *EGFR* (A) and *KRAS* (B) mutations. ^*^ All exon 20 T790M *EGFR* mutations were detected in combination with either an exon 19 deletion or an exon 21 L858R mutation

Among the *EGFR* mutations, deletions in exon 19 were the most common (39; 52.7 %), followed by the L858R point mutation in exon 21 (21; 28.4 %) (Fig. 1). Besides these regular mutations, one insertion (V769-D770 ins ASV) and four different point mutations in exon 20 (S768I (twice), S768N, G779S, and H805Y), and four different point mutations in exon 21 (R831H, V845A, D855N and L861Q (twice)) were found. Also, three (TKI resistant) T790M point mutations in exon 20 were detected, all in combination with another (activating) *EGFR* mutation (one deletion in exon 19 and two L858R point mutations in exon 21). The single nucletoid polymorphism A840T in exon 21 was detected twice.

### *KRAS* mutations

*KRAS* mutation status was determined in 873 samples (699 ADC, 42 SCC, 132 others), 845 of which could be interpreted. Of these samples, 497 were derived from primary tumors and 363 from different metastatic sites. For 13 pleural biopsies/excisions, distinction between a primary tumor and a metastasis was not possible. The samples were derived from 860 individual patients, of whom there was an interpretable result in 832 patients. For 13 patients the mutation status of both their primary tumor and a metastasis was determined. These patients were considered *KRAS* mutation positive if either the primary tumor or the metastasis was tested positive. *KRAS* mutations were detected in 277 (33.3 %) patients, 244 of whom had a diagnosis of ADC (frequency in ADC 36.9 %). Like *EGFR* mutations, *KRAS* mutations were significantly more common in ADC than in SCC or other tumor types (36.9 %, 2.5 %, and 24.6 % respectively, *p* < 0.001) and there was a trend towards more mutations in female compared to male patients (36.4 % vs. 30.6 %, *p* = 0.090), but this did not reach statistical significance (Table 1). The smoking history could be obtained for 287 patients with a known *KRAS* mutation status. There was a lower *KRAS* mutation frequency in never smokers compared to former and current smokers (12.0 % vs. 33.9 % vs. 36.6 % respectively, *p* = 0.044). There was no difference in average number of packyears between *KRAS* mutation positive and *KRAS* mutation negative patients (30.7 vs. 29.9, *p* = 0.808). The *KRAS* mutations consisted of point mutations in codon 12 (239; 86.3 %), codon 13 (20; 7.2 %) and codon 61 (18; 6.5 %) (Fig. 1).

### Combined *EGFR* and *KRAS* mutations

For 752 patients both the *EGFR* and *KRAS* mutation status were determined. *EGFR* and *KRAS* mutations were mainly found mutually exclusive. Only two cases (0.3 %) of combined *EGFR* and *KRAS* mutations were detected: *EGFR* exon 20 S768N combined with *KRAS* exon 2 G12A and *EGFR* exon 20 G779S combined with *KRAS* exon 2 G12C. These were both cases of ADC in males, detected in bronchial biopsies.

### Primary carcinomas versus metastases

The difference in mutation frequency between primary tumors and metastases was studied. Because nearly all *EGFR* and *KRAS* mutations were found in ADC, we performed these calculations for ADC only. Pleural biopsies and excisions were excluded from these calculations, since discrimination between outgrowth of a primary tumor or a metastasis was not always feasible. The *EGFR* status was determined in 360 primary tumors and 261 metastatic sites, the *KRAS* status in 394 primary tumors and 268 metastatic sites. For *EGFR* mutations, no statistically significant difference in frequency in primary tumors compared to metastases (11.4 % vs. 10.0 %, *p* = 0.602) was observed (Table 2), whereas for *KRAS* the mutation frequency was significantly higher in primary tumors than in metastases (40.1 % vs. 31.3 %, *p* = 0.026) (Table 3).

**Table 2:**
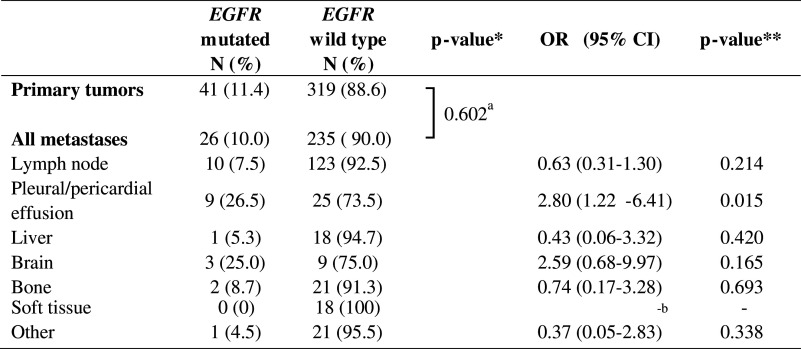
Frequency of *EGFR* mutations in primary tumors and metastases

*OR* odds ratio; *CI* confidence interval

*P-value calculated by Fisher’s exact test

**P-value calculated by binary logistic regression analysis

^a^Primary tumors vs. all metastases combined; *p* = 0.034 for primary tumors vs. different tumor locations separately

^b^Can not be calculated because 0 *EGFR* mutations were detected in this category

**Table 3:**
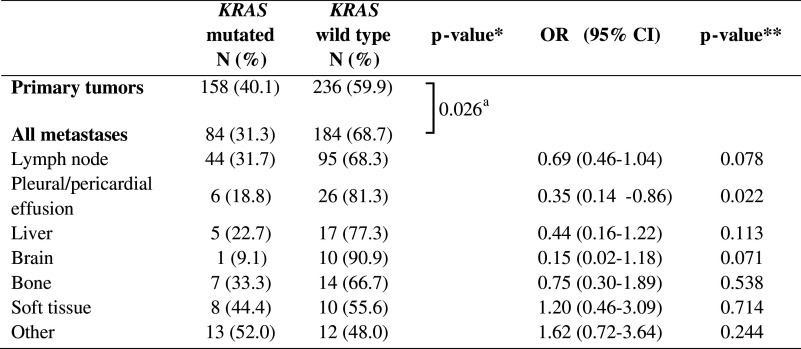
Frequency of *KRAS* mutations in primary tumors and metastases

*OR* odds ratio; *CI* confidence interval

*P-value calculated by Fisher’s exact test

**P-value calculated by binary logistic regression analysis

^a^Primary tumors vs. all metastases combined; *p* = 0.017 for primary tumors vs. different tumor locations separately

Next, we divided the metastasis locations in different tissue types, using main categories with at least 10 samples each. The categories were lymph node, pleural and pericardial effusion, liver, brain, bone, soft tissue and others. The results are summarized in Tables 2 and 3. Overall a significant difference between different categories was observed for the presence of both *EGFR* and *KRAS* mutations (*p* = 0.034 and *p* = 0.017, respectively). Binary logistic regression analysis was performed to test which category or categories was responsible for this difference. Both for *EGFR* and *KRAS* the category pleural and pericardial effusions was associated with a difference in mutation frequency compared to primary tumors (*EGFR* OR = 2.80 (95 % CI 1.22-6.41), *p* = 0.015; *KRAS* OR = 0.35 (95 % CI 0.14–0.86), *p* = 0.022). The frequency of *EGFR* mutations in this category was 26.5 %; the frequency of *KRAS* mutations was 18.8 %. Also brain metastases showed a trend towards an increase in *EGFR* mutation frequency and a decrease in *KRAS* mutation frequency (25.0 % and 9.1 %, respectively) compared to the group of primary tumors, but this did not reach statistical significance.

The present study population contained 13 pairs of matched primary tumors and metastases. There was discordance of the *EGFR* and/or *KRAS* mutation status in 4 out of these 13 (30.8 %) pairs, leading to a clinically discordant mutation status in 3/13 (23.1 %) pairs: one *KRAS* mutation was present in the metastasis but not in the primary tumor; one *EGFR* mutation was present in the metastasis but not in the primary tumor, while this primary tumor showed a *KRAS* mutation that was not present in the metastasis; and one primary tumor showed both a deletion in exon 19 and the T790M point mutation in exon 20 of *EGFR*, whereas the metastasis only showed the deletion in exon 19. In one pair the primary tumor and the metastasis each contained a different *KRAS* mutation (exon 1 G12V and G12R, respectively).

## Discussion

To our best knowledge this is the first study showing an association between *EGFR* mutation rate and metastatic location in patients with lung adenocaricnoma in the European population. We found an increased *EGFR* mutation frequency (27 %) and a decreased *KRAS* mutation frequency (19 %) in patients with malignant pleural or pericardial effusions. A similar trend was present in patients with brain metastases, although this did not reach statistical significance. These results correspond with two studies from East Asia, showing significantly higher *EGFR* mutation rates in tumors with malignant pleural effusions compared to those without [12], and a relatively high *EGFR* mutation rate in brain metastases [13]. These observations are important for the treatment of patients with metastasized ADC of the lung. In patients with malignant pleural or pericardial effusion, the treating physician could make an extra effort to obtain tissue for mutation analysis or, if no tissue can be obtained, a test treatment with TKI could be considered. The genetic profile of a tumor determines its biological behavior and the *EGFR* and *KRAS* mutation status could very well influence the pattern of metastasis formation, a topic that merits further investigation.

Several studies have shown discordance in the presence of *EGFR* and *KRAS* mutations between primary tumors and matched metastases [14, 15]. In the 13 pairs of primary tumors and matched metastases in our cohort there were different mutations in 4 out of 13 (31 %) pairs, leading to a clinically different mutation status in 3/13 (23 %) pairs. In these latter cases the difference in mutation status between primary tumor and metastasis could have clinical implications in view of the response to treatment with TKIs. We do not have information concerning the treatment of these patients with TKIs or chemotherapy between the times at which the primary tumor and metastasis samples were obtained. Treatment could have caused selection pressure, leading to a growth advantage for certain clones, thus giving rise to a different mutation status. In the process of metastasis formation, outgrowth of certain clones of tumor cells can also take place without the influence of selection pressure.

The overall frequency of *EGFR* and *KRAS* mutations has mainly been studied in East Asian and North American and to a lesser extent in South European populations, showing large differences between these populations. This study is the first to address this topic in a large West European cohort. In adenocarcinomas, we found *EGFR* mutations in 11 % of cases. This is far below the 36–66 % reported for East Asia [2, 3], but lies within the range of 6–24 % reported for North America [4, 5], 6–16 % for Southern Europe [9, 16], and 11 % in a recent smaller North European (Norwegian) study [17]. This similarity could be explained by the fact that the ethnic background of our study population will probably correspond closer to the North American and South European than to the East Asian population, though environmental influences may still differ between these populations. Exon 18 of the *EGFR* gene was not included in the analysis, because this exon was not routinely investigated in all participating laboratories. This may have led to an underestimation of the *EGFR* mutation frequency, although exon 18 mutations have been reported to account for only a small minority of total *EGFR* mutations [4, 18].

*KRAS* mutations were detected in 37 % of ADC, far above the 2–9 % reported for East Asia [3, 6]. The *KRAS* mutation frequency in our West European cohort is even higher than the 12–31 % and 11–29 % reported for North America [4, 8] and Southern Europe [7, 9], respectively. The observed KRAS mutation frequency is close to the 39 % reported in a Dutch validation study for HRM that analyzed a small group of lung cancer patients [19]. A possible partial explanation for the observed difference could be the fact that in most studies only point mutations in codons 12 and 13 were studied, which make up over 90 % of *KRAS* mutations [20]. In addition to codons 12 and 13, we also studied codon 61 and a substantial 7 % of mutations were detected in this codon, which would have been missed if only the hotspots codons 12 and 13 had been tested.

*EGFR* mutations were twice as often observed in female compared to male patients (13 % vs. 6 %, *p* < 0.001). This observation confirms that the difference in *EGFR* mutation frequency between male and female patients that previously has been described in Asian and South European populations, is also present in the West European population [2, 3, 9, 16]. We found a trend towards more *KRAS* mutations in female compared to male patients (36 % vs. 31 %), but this did not reach statistical significance. Previous studies also did not show a statistically significant correlation between *KRAS* mutation status and sex, though some studies showed a trend towards more mutations in males [6, 21]. However, in these studies the total number of *KRAS* mutations was very low as these were performed in East Asian populations.

The *EGFR* mutation frequency was over five times higher in never smokers compared to former and current smokers. This strong negative association with smoking history has also been observed in Asian, North American, and South European populations [2, 3, 5, 9, 16]. The association between presence of *KRAS* mutations and smoking has also been reported before [22].

Both *EGFR* and *KRAS* mutations were present more commonly in ADC compared to SCC and other tumor types. This observation confirms previous results [16].

The *EGFR* mutations consisted mainly of deletions in exon 19 and the L858R point mutation in exon 21. These two types of mutations combined formed 81 % of all *EGFR* mutations in our cohort, which approximates the 85 to 90 % mentioned in previous studies [23]. Possibly the proportion of these two types of mutations is somewhat lower in the present study, because some of the other studies only investigated the more common mutation sites. Besides two L861Q point mutations in exon 21, known to be TKI-sensitive [23], we found 7 different more rare point mutations: S768I (twice), S768N, G779S, and H805Y in exon 20 and R831H, V845A, and D855N in exon 21. All of these rare point mutations have been reported before [14, 23–27], except for the H805Y and V845A mutations, though other mutations at the same position have been reported [28, 29]. For all these point mutations, the clinical significance and sensitivity to TKIs are unknown.

Three T790M point mutations in exon 20, known to cause resistance to treatment with TKIs, were detected, all in combination with an activating *EGFR* mutation (one deletion in exon 19 and two L858R point mutations in exon 21). The T790M point mutation is considered to cause secondary resistance to treatment with TKIs and thus is expected to be present after treatment only [30], although others report it to be present in tumors before treatment [31]. The insertion in exon 20 that was detected, has been reported previously [3, 6]. This and other insertions in exon 20 have been associated with TKI resistance [23, 32].

*EGFR* and *KRAS* mutations are generally considered to be mutually exclusive [2–4, 9], though there are some studies showing combined *EGFR* and *KRAS* mutations [33]. We found 2 cases of combined *EGFR* and *KRAS* mutations. Interestingly, in both cases the *EGFR* mutation was a rare point mutation (S768N and G779S) with unknown clinical significance. Possibly these mutations do not cause constitutional activation of EGFR and thus have no clinical significance. This could also explain why they have often been found in combination with known activating *EGFR* mutations, as described previously [24, 26].

A limitation of the present study is the fact that it does not provide a true cross-section of non-small cell lung carcinomas, since most analyses were requested by the treating physician. This introduces a selection bias towards more advanced disease stages, because the treating physician will generally not request the analysis for patients who have been cured surgically. However, this corresponds to daily clinical practice, and the population studied here, is the population that would potentially benefit from treatment with TKIs, which still makes the data relevant.

In conclusion, *EGFR* and *KRAS* mutation frequencies are not evenly distributed among different metastatic sites, with a relative increase in *EGFR* mutation frequency and a decrease in *KRAS* mutation frequency in pleural and pericardial effusions compared to primary tumors, which could have implications for patient management. In the West European Dutch population the overall *EGFR* mutation frequency lies within the range of frequencies described for North American and South European populations. For *KRAS* the frequencies are considerably higher than those described in any other population studied to date. Both *EGFR* and *KRAS* mutations were found more often in ADC than in other tumor types and *EGFR* mutations were more common in females compared to males.
